# The Development of a Wearable-Based System for Detecting Shaken Baby Syndrome Using Machine Learning Models

**DOI:** 10.3390/s25154767

**Published:** 2025-08-02

**Authors:** Ram Kinker Mishra, Khalid AlAnsari, Rylee Cole, Arin Nazarian, Ilkay Yildiz Potter, Ashkan Vaziri

**Affiliations:** 1 BioSensics LLC, Newton, MA 02458, USA; ram.mishra@biosensics.com (R.K.M.); rylee.cole@biosensics.com (R.C.); arin.nazarian@icloud.com (A.N.); ilkay.yildiz@biosensics.com (I.Y.P.); 2 Department of Emergency Medicine, Sidra Medicine, Al Rayyan Road, Doha P.O. Box 26999, Qatar; kalansari@sidra.org; 3 Departments of Clinical Emergency and Clinical Pediatrics, Weill Cornell Medicine, 8C9R+735, Education City, Al Luqta St, Doha P.O. Box 24144, Qatar; 4 Department of Clinical Pediatrics, Qatar University, Doha P.O. Box 2713, Qatar

**Keywords:** Shaken Baby Syndrome, wearable sensor, inertial measurement unit, machine learning

## Abstract

Shaken Baby Syndrome (SBS) is one of the primary causes of fatal head trauma in infants and young children, occurring in about 33 per 100,000 infants annually in the U.S., with mortality rates being between 15% and 38%. Survivors frequently endure long-term disabilities, such as cognitive deficits, visual impairments, and motor dysfunction. Diagnosing SBS remains difficult due to the lack of visible injuries and delayed symptom onset. Existing detection methods—such as neuroimaging, biomechanical modeling, and infant monitoring systems—cannot perform real-time detection and face ethical, technical, and accuracy limitations. This study proposes an inertial measurement unit (IMU)-based detection system enhanced with machine learning to identify aggressive shaking patterns. Findings indicate that wearable-based motion analysis is a promising method for recognizing high-risk shaking, offering a non-invasive, real-time solution that could minimize infant harm and support timely intervention.

## 1. Introduction

Shaken Baby Syndrome (SBS), also referred to as Abusive Head Trauma (AHT), is a leading cause of fatal head injuries in infants and young children [1,2]. SBS occurs when a caregiver forcefully shakes a child, leading to intracranial bleeding, retinal hemorrhages, and severe brain damage [3]. It is estimated that SBS affects 33 cases per 100,000 infants annually in the United States, with mortality rates ranging from 15% to 38% [4,5]. Survivors often suffer from permanent disabilities, including cognitive impairments, blindness, and motor dysfunction [6,7,8,9].

Detecting SBS remains a significant challenge due to the absence of external injuries and its delayed clinical presentation. Current detection methodologies include neuroimaging techniques such as computed tomography (CT) and magnetic resonance imaging (MRI), which can identify intracranial injuries post-factum [10]. While these imaging methods are crucial for confirming SBS cases, they are not preventive or real-time detection tools. Additionally, biomechanical models have been developed to study the kinematics of SBS, but their clinical application remains limited due to ethical and technical constraints [11].

Several monitoring systems have been proposed to detect abusive shaking events. Some infant monitoring devices utilize accelerometers embedded in smart baby cribs and wearable systems to track sudden movements [12]. However, these systems primarily detect gross motor movements and lack specificity in distinguishing between normal infant care activities (such as rocking or burping) and abusive shaking. Video-based monitoring systems provide an additional method for detecting suspicious caregiver behavior, but they require constant surveillance and can be hindered by occlusions and privacy concerns [13].

Having reliable tools to detect SBS is expected to lead to a decrease in the syndrome’s prevalence when used in high-risk groups, specifically being used for extra evidence to identify abusers and provide valid information to facilitate family reunion. The need for an accurate and real-time SBS detection system has led researchers to explore inertial measurement unit (IMU)-based solutions. IMU sensors, consisting of accelerometers and gyroscopes, can quantify motion patterns with high temporal resolution. By integrating machine learning algorithms, these systems can classify movement characteristics indicative of SBS while differentiating them from benign infant care activities. Machine learning models, such as decision trees and neural networks, can analyze sensor data and provide real-time alerts for high-risk movements [14].

This study aims to develop an IMU-based detection system augmented with machine learning to classify aggressive shaking events. By leveraging wearable sensor technology and advanced classification models, this system provides a non-invasive and real-time solution for SBS detection, potentially reducing harm to infants and enabling early intervention. The work demonstrates the expanding potential of combining wearable sensor data with artificial intelligence to create unobtrusive health assessment tools and complement previous efforts in this area related to predicting disease activities [15,16], continuous cardiorespiratory monitoring [12], and biometric identification [17].

## 2. Materials and Methods

### 2.1. Study Overview

**Figure 1:**
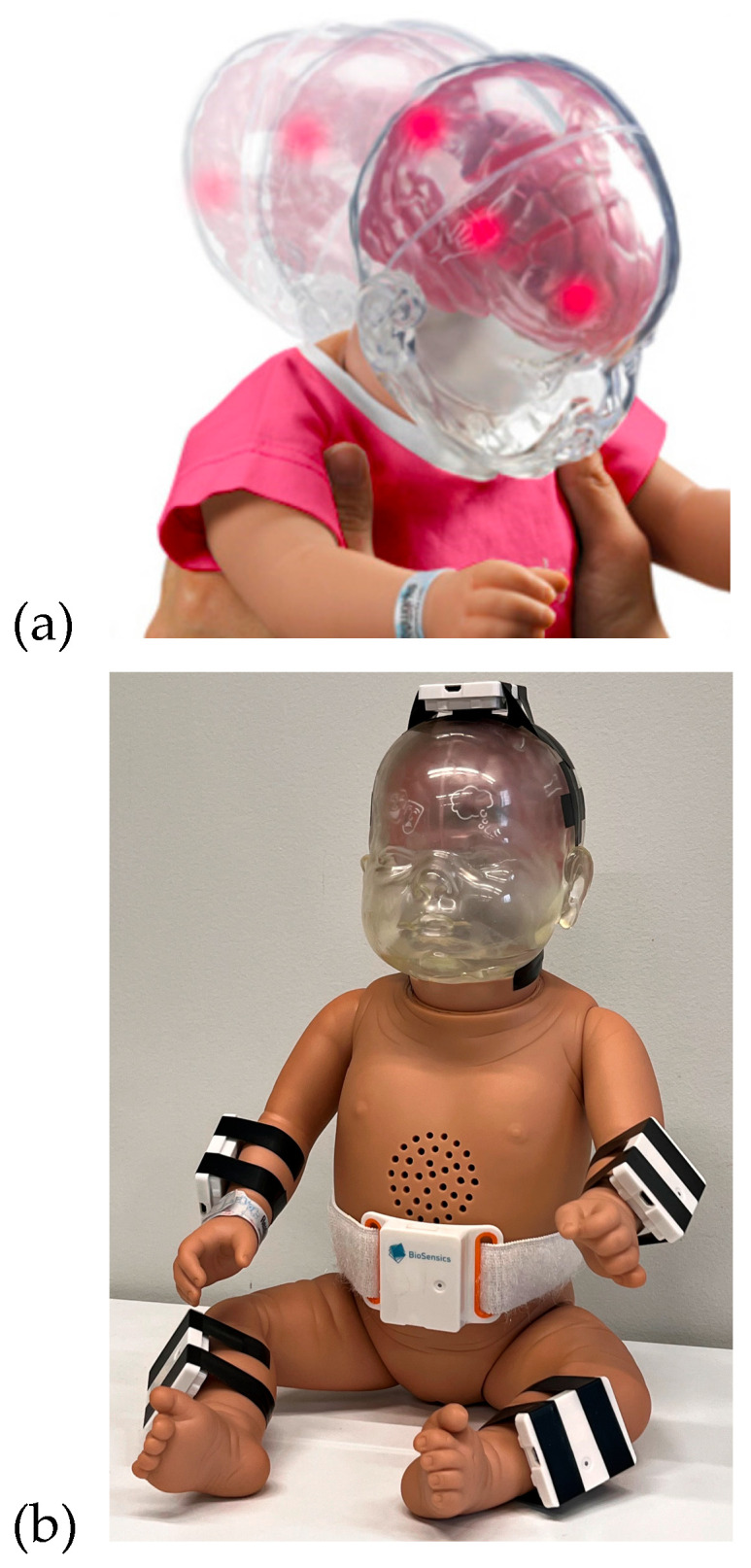
(**a**) RealCare™ Shaken Baby Doll manufactured by RealityWorks. (**b**) The doll was equipped with 6 IMU sensors.

**Figure 2:**
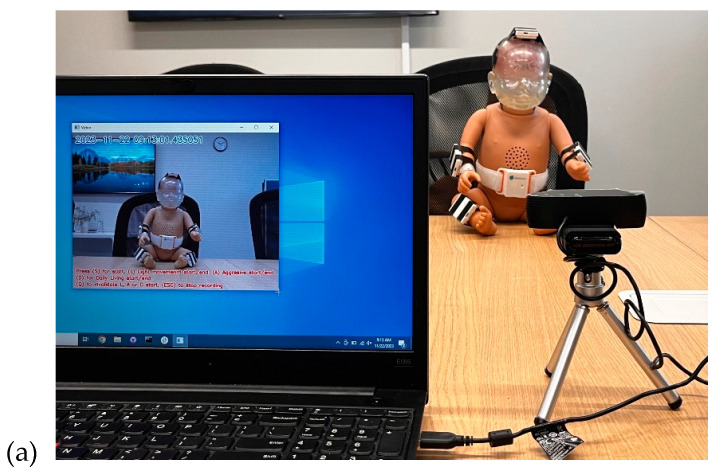

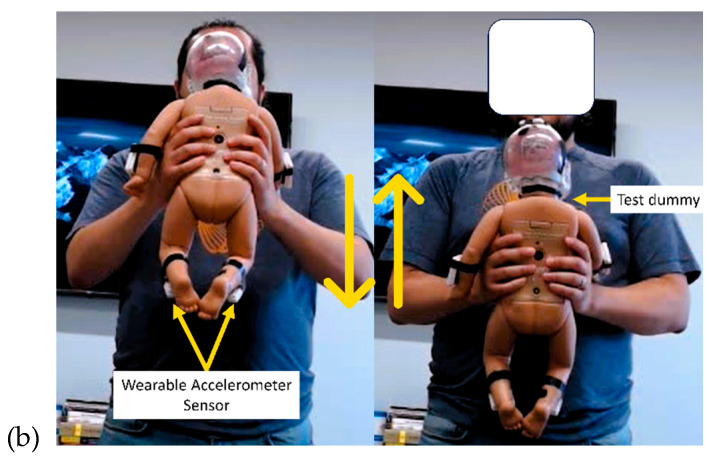
(**a**) The SBS recording setup, including a laptop with an annotation app, a camera, and a RealCare™ Shaken Baby Doll equipped with sensors. (**b**) Volunteer performing shaking movements in different positions using the test dummy.

In this study, we developed a wearable-based system utilizing IMU sensors and a machine learning approach to detect aggressive shaking movements indicative of Shaken Baby Syndrome. Data was collected using the RealCare™ Shaken Baby Doll (RealityWorks, Eau Claire, WI, USA), a validated surrogate designed to simulate realistic infant movements and provide visual feedback when exposed to high acceleration forces. The doll was equipped with six proprietary IMU sensors (BioSensics, Newton, MA, USA), each containing a 9-axis accelerometer and gyroscope, strategically placed on the head, chest, wrists, and shins to capture full-body motion dynamics (Figure 1). These sensors recorded high-frequency (100 Hz) motion data across multiple shaking scenarios. A proprietary video labeling software (BioSensics, Newton, MA, USA) was developed to ensure precise synchronization between recorded video and sensor data, allowing for accurate annotation of different movement types, including aggressive shaking, light shaking, and daily infant care activities. The data collection process followed a structured protocol, where each participant performed a predefined sequence of 18 tasks, including 13 shaking movements and 5 simulated caregiving activities (Figure 2). Tasks were classified into “Light Movement” and “Aggressive Movement” categories based on acceleration thresholds and visual indicators from the doll’s LEDs. Real-time signal monitoring was implemented to ensure data reliability, allowing for immediate verification of sensor integrity and synchronization. Collected data were stored in a HIPAA-compliant system for further analysis. This setup provided a comprehensive dataset for developing machine learning algorithms capable of real-time SBS detection, ensuring a robust and interpretable detection model.

### 2.2. Study Hardware

RealCare™ Shaken Baby Dolls (RealityWorks, Eau Claire, WI, USA) were used for this study. They are designed to demonstrate movements that could potentially pose harm to infants. This baby doll is equipped with a sequence of LEDs located on its head, which illuminate upon reaching a specific acceleration threshold, as shown in Figure 1 (top). These visual cues alert the users that the movement may pose a risk of causing potential harm to a baby. The RealCare Shaken Baby Doll has been used in multiple studies and educational and preventative programs [18,19,20,21]). The baby doll used in this study weighed 7 lbs and was 21 in tall.

LEGSys™ IMU Sensors (BioSensics, Newton, MA, USA) were used for the study. These sensors are equipped with a 9-axis IMU that integrates accelerometer and gyroscope data. Sensors sample data at a high frequency of 100 Hz. A total of 6 sensors were attached to the doll at the following locations, as shown in Figure 1 (bottom):

- 2 sensors on wrists (1 on each wrist);
- 2 sensors on shins (1 on each shin);
- 1 sensor on top of the navel;
- 1 sensor on top of the head/cranial apex.

### 2.3. Experimental Design

The arrangement for data collection is depicted in Figure 2. The entire process of data collection was recorded via video. At the initiation of each session, the sensors were activated and synchronized with the ongoing video recording. Each task was executed with a consistent duration of 15 s. Depending on the intensity level, each test condition was categorized as either “Light Movement” or “Aggressive Movement.” IMU sensors, affixed to the doll, captured both the acceleration and angular velocity experienced by the doll during each task. To ensure adequate acceleration levels were attained during high-intensity tasks, the baby doll’s LEDs illuminated once a specific acceleration threshold was reached. This real-time feedback mechanism prompted participants to escalate their agitation during high-intensity (i.e., aggressive shaking) tasks, ultimately serving as the marker for detecting the activity as “Aggressive Movement”.

A proprietary software (BioSensics, Newton, MA, USA) was developed to facilitate precise data labeling during the experimental process. With this software, investigators have the capability to record high-quality video footage while seamlessly annotating the start and end points of specific events by simply clicking a button. This feature also enables the reporting of an error if an unwanted event occurs (i.e., mistakenly giving wrong instructions). This user-friendly interface ensures efficient and accurate data labeling, particularly in the context of distinguishing between aggressive shaking, light shaking, and simulated activities of daily care of the RealCare^TM^ Baby Doll.

The video recordings were annotated in real-time using a proprietary software application (version 1.0) used to mark the start and end of each test condition, labeling the type of activity performed (i.e., Aggressive Movement, Light Movement, or daily care), which provided the ground truth for the development of the machine learning model.

To ensure the model could reliably distinguish harmful shaking from normal caregiving, the study protocol intentionally incorporated a range of everyday infant care activities—such as rocking, burping, arm swinging, and diaper changing. These benign movements reflect realistic handling scenarios and were essential for training the model to recognize non-aggressive interactions commonly encountered in caregiving environments. The prescribed activities were categorized into shaking movements and simulated activities of daily care, listed in Table 1 below:

### 2.4. Data Acquisition

Eight volunteers (31.7 ± 10.2 years old, 2 female) performed a series of 18 tasks involving shaking movements and simulated activities of daily care based on the data collection procedure outlined in the Experimental Design subsection. Each participant performed a series of 18 tasks involving (1) shaking movements to simulate shaking a baby that could potentially result in SBS (a total of 13 tasks) and (2) simulated activities of daily care (a total of 5 tasks), as shown below. All tasks were recorded and labeled using the video labeling software.

The research coordinator followed a predefined research protocol and provided clear and precise instructions to the participants. This included explaining how each task should be performed, emphasizing the importance of positions of holding the baby, and ensuring that participants understood the study’s objectives.

The coordinator conducted sensor testing to prevent data loss due to technical malfunctions. This involved checking the functionality of the IMU sensors, confirming that they were accurately measuring the doll’s movements and that sensor batteries were fully charged.

To maintain consistency in data collection, the research coordinator ensured that the environment in which the tasks were performed remained constant. Factors such as performing the task while standing or sitting remained consistent, and background noise was controlled to minimize potential distractions that could affect data quality. Before data collection, the research coordinator ensured the sensors were correctly attached and calibrated to capture precise movement data during each task.

During the task, the research coordinator could see the sensor signal using the in real time on a laptop. Lastly, the collected data was stored in a HIPAA-compliant Box folder to maintain data integrity and confidentiality. This ensured that sensitive information related to the participants and their activities was secure and compliant with healthcare data privacy regulations. Overall, 144 tasks were performed, including 56 aggressive tasks and 88 non-aggressive tasks. Sensor recordings from 6 different body locations yielded 864 recordings.

### 2.5. Data Analysis

A structured and interconnected sequence of data processing and analysis steps that collectively form a data processing workflow was developed. The raw acceleration data from sensors was offloaded and analyzed offline. No filtering or outlier detection was performed to avoid introducing latency in real-time detection.

Step 1. A sliding window segmentation approach was used to break the signal into segments of 3 s with an overlap of 1 s. The window size of 3 s allowed for the assessment of short-term patterns and activities, while the 1 s overlap ensured that no information was lost between adjacent windows.

**Figure 3:**
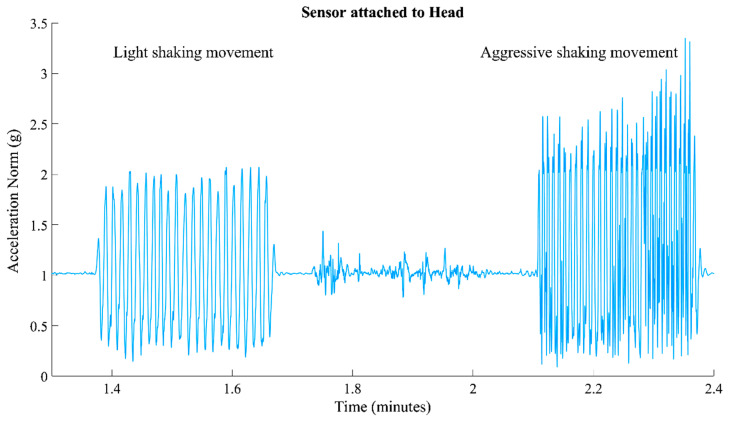
Illustration of the acceleration signal’s norm, depicting a sequence starting with light shaking movement and transitioning into Aggressive Movement.

Step 2. The acceleration norm was estimated for each segment using the acceleration signals of the three axes. Figure 3 shows the acceleration signal’s norm corresponding to light and aggressive shaking movements. The norm of acceleration is the magnitude of acceleration, calculated as

$$Norm=\;\sqrt{a_{x}^{2}+\;a_{y}^{2}+a_{z}^{2}}$$

where $a_{x}$, $a_{y}$, and $a_{z}$ are the accelerations along the x, y, and z axes, respectively. The data can be highly dependent on the device’s orientation when using individual accelerometer axes (e.g., x, y, and z). Estimating the norm eliminates this orientation dependency, allowing for consistent interpretation and threshold setting for movement detection.

Step 3. We conducted feature extraction on each segment to gather pertinent details regarding the movement pattern. We computed descriptive statistics, encompassing mean; standard deviation; percentiles at the 10th, 50th, and 95th positions; minimum; maximum; skewness; kurtosis; root mean square (RMS); average frequency; and entropy. These features were chosen due to their low computational complexity.

**Figure 4:**
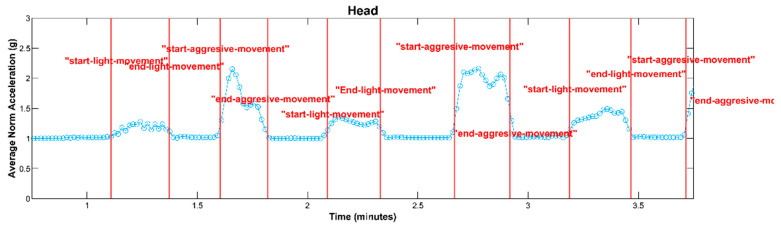
An illustration depicting the average of the acceleration signal’s norm, showcasing the classification of movements into the “Aggressive Movement” and “Light Movement” categories, with marker lines denoting the start and end points of each classified movement event.

Step 4. Once these events were identified in the video recordings, the next step was to confirm their alignment with the accelerometer data recorded by the wearables. This confirmation ensured that the sensor data accurately represented the observed events. The class labels “Light Movement” and “Aggressive Movement” were reviewed by a team member using the recorded video and signal together (Figure 4). This meticulous process ensured that the collected data was accurately labeled and could be used effectively for subsequent analysis and research purposes.

## 3. Results

Machine learning-based models were developed to classify each segment as “Aggressive Movement” or “Non-Aggressive Movement” based on the input features. A decision tree automatically identified the relevant features (independent variables) likely to impact the classification. Employing a recursive process, the decision tree algorithm divided the data into subsets based on feature values, forming a tree-like structure. At each node of this structure, the decision tree selected the optimal feature for data partitioning, employing the Gini impurity as the criterion [22].

Overall, 5092 segments of 3 s were obtained, with 749 segments labeled as Aggressive Movement. We allocated 70 percent of the data for training our decision tree model, reserving the remaining 30 percent for evaluating the model’s performance. The tree continued to split until it reached a maximum depth of 2.

**Figure 5:**
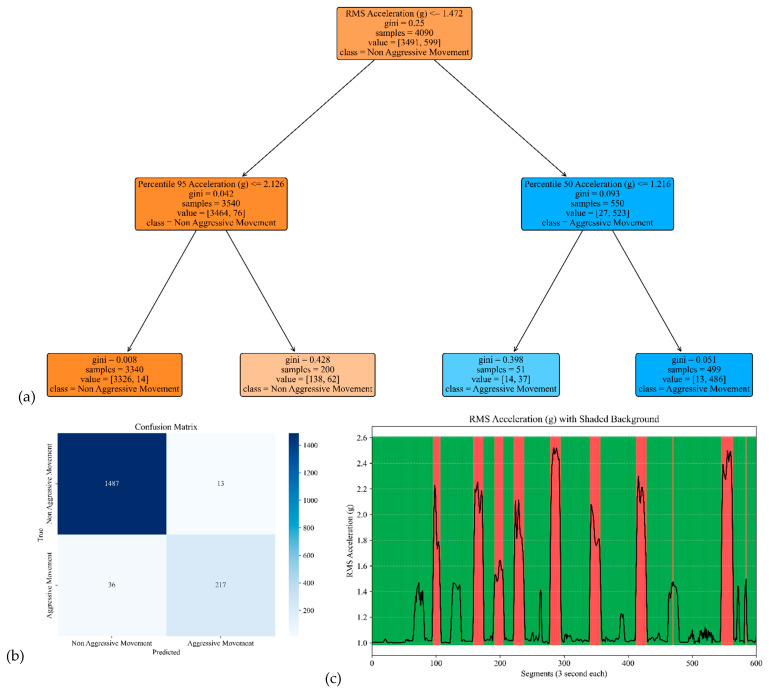
The model performance of the sensor attached to the head. (**a**) The analysis identifies pivotal classification features, with the root mean square (RMS) of the acceleration signal’s norm taking precedence, followed by the 95th percentile of the acceleration’s norm and the median of the acceleration signal’s norm. (**b**) The confusion matrix included True vs. Predicted Negative (Non-Aggressive) and Positive (Aggressive) classes. (**c)** The RMS was estimated for each segment of 3 s of the acceleration’s norm. Red shading represents segments identified as Aggressive Movement, and green shading represents Non-Aggressive Movement.

The model performance of the sensor attached to the head is shown in Figure 5. The model yielded an accuracy, precision, specificity, and sensitivity of 0.97, 0.94, 0.99, and 0.86, respectively. These metrics indicate that the model achieved high overall accuracy and precision, distinguishing between Aggressive and Non-Aggressive Movements. The confusion matrix included True Negatives (TNs), where 1487 instances were correctly classified as “Non-Aggressive Movement;” True Positives (TPs), where 217 instances were correctly classified as “Aggressive Movement;” False Negatives (FNs), where 36 instances of “Aggressive Movement” were incorrectly classified as “Non-Aggressive Movement.”; and False Positives (FPs), where 13 instances of “Non-Aggressive Movement” were incorrectly classified as “Aggressive Movement.” These results demonstrate the model’s ability to make accurate predictions, effectively distinguishing between the two classes while minimizing False Positives and Negatives.

**Figure 6:**
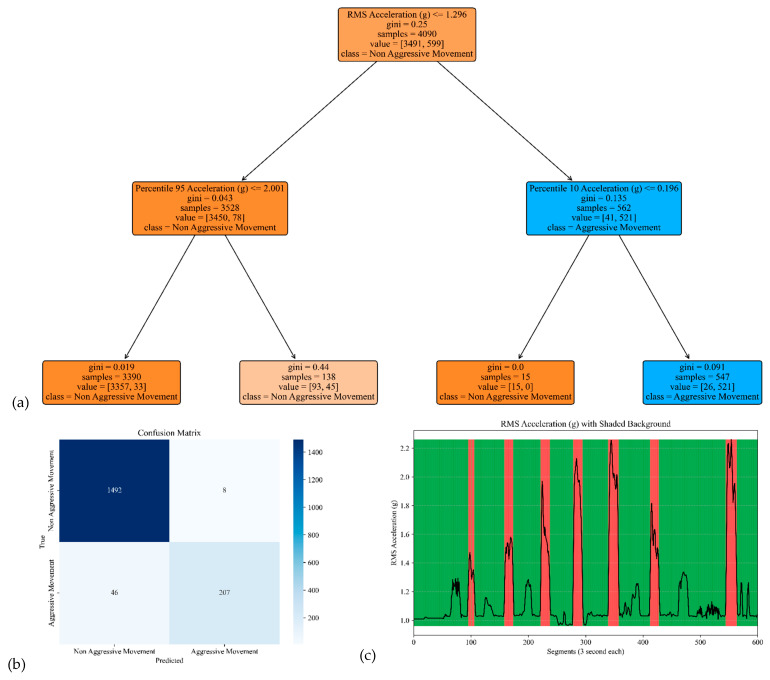
The model performance of the sensor attached to the chest. (**a**) The analysis identifies pivotal classification features, with the root mean square (RMS) of the acceleration signal’s norm taking precedence, followed by the 95th percentile of the acceleration’s norm and the 10th percentile of the acceleration signal’s norm. (**b**) The confusion matrix included True vs. Predicted Negative (Non-Aggressive) and Positive (Aggressive) classes. (**c**) The RMS was estimated for each segment of 3 s of Acceleration’s norm. Red shading represents segments identified as Aggressive Movement, and green shading represents Non-Aggressive Movement.

The model performance of the sensor attached to the chest is shown in Figure 6. The corresponding model yielded an accuracy, precision, specificity, and sensitivity of 0.97, 0.96, 0.99, and 0.82, respectively (see Table 2).

**Figure 7:**
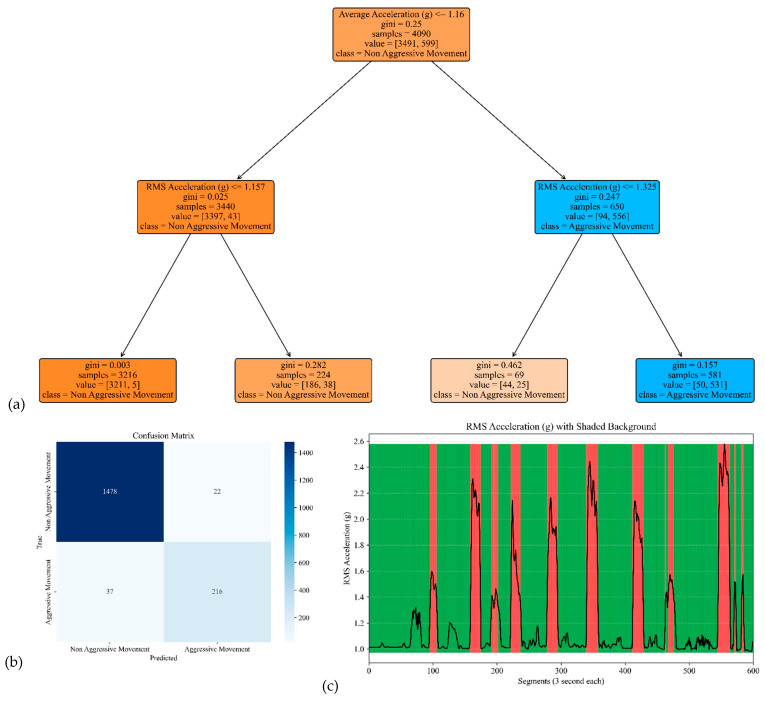
The model performance of the sensor attached to the left arm. (**a**) The analysis identifies pivotal classification features, with the average of the acceleration signal’s norm taking precedence, followed by the RMS of the acceleration’s norm of the acceleration signal’s norm. (**b**) The confusion matrix included True vs. Predicted Negative (Non-Aggressive) and Positive (Aggressive) classes. (**c**) The RMS was estimated for each segment of 3 s of the acceleration’s norm. Red shading represents segments identified as Aggressive Movement, and green shading represents Non-Aggressive Movement.

**Figure 8:**
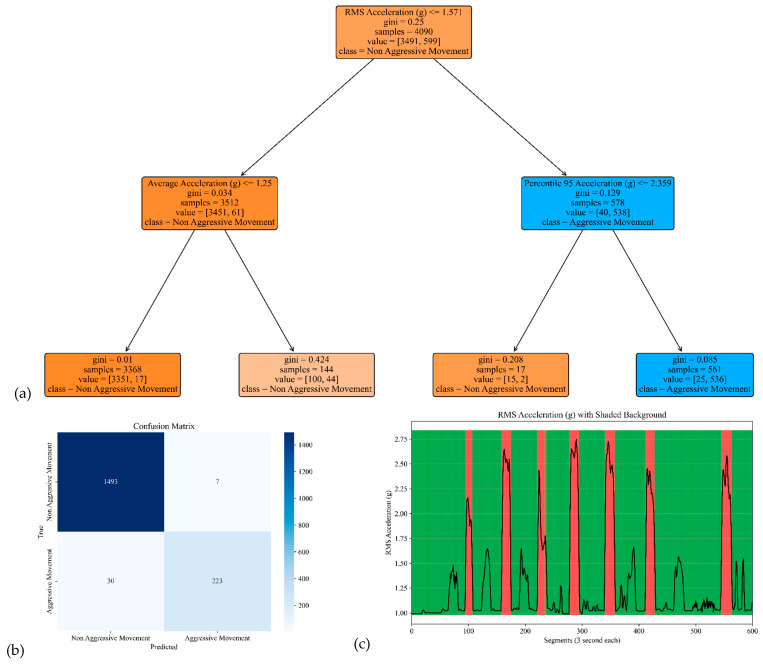
The model performance of the sensor attached to the right arm. (**a**) The analysis identifies pivotal classification features, with the RMS of the acceleration signal’s norm taking precedence, followed by the average and 95th percentile of the acceleration’s norm. (**b**) The confusion matrix included True vs. Predicted Negative (Non-Aggressive) and Positive (Aggressive) classes. (**c**) The RMS was estimated for each segment of 3 s of the acceleration’s norm. Red shading represents segments identified as Aggressive Movement, and green shading represents Non-Aggressive Movement.

The model performances for the sensor attached to the left and right arms are shown in Figure 7 and Figure 8. The corresponding model yielded an accuracy, precision, specificity, and sensitivity of 0.97, 0.91, 0.99, and 0.85, respectively, for the left arm and 0.97, 0.93, 0.99, and 0.85, respectively, for the right arm (see Table 2).

**Figure 9:**
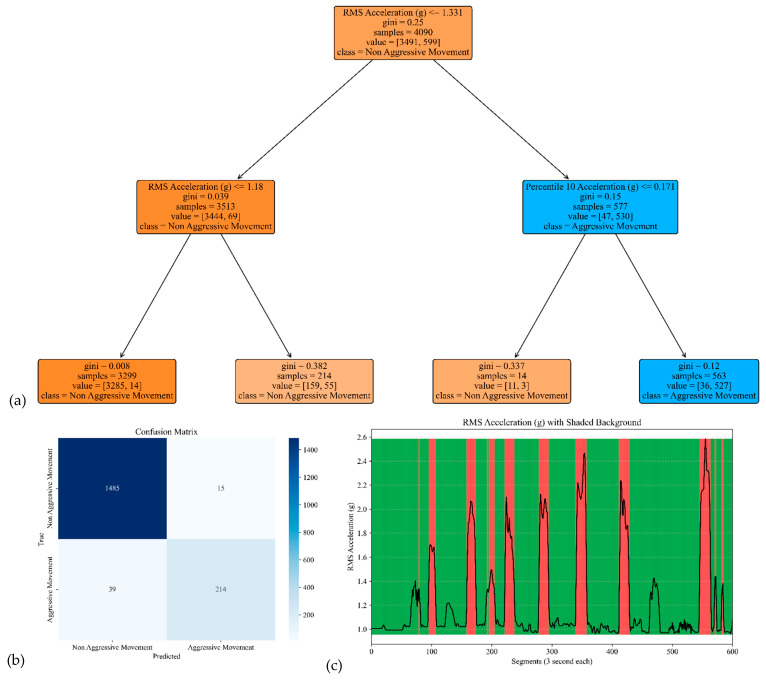
The model performance of the sensor attached to the left leg. (**a**) The analysis identifies pivotal classification features, with the RMS of the acceleration signal’s norm taking precedence, followed by the 10th percentile of the acceleration’s norm. (**b**) The confusion matrix included True vs. Predicted Negative (Non-Aggressive) and Positive (Aggressive) classes. (**c**) The RMS was estimated for each segment of 3 s of the acceleration’s norm. Red shading represents segments identified as Aggressive Movement, and green shading represents Non-Aggressive Movement.

**Figure 10:**
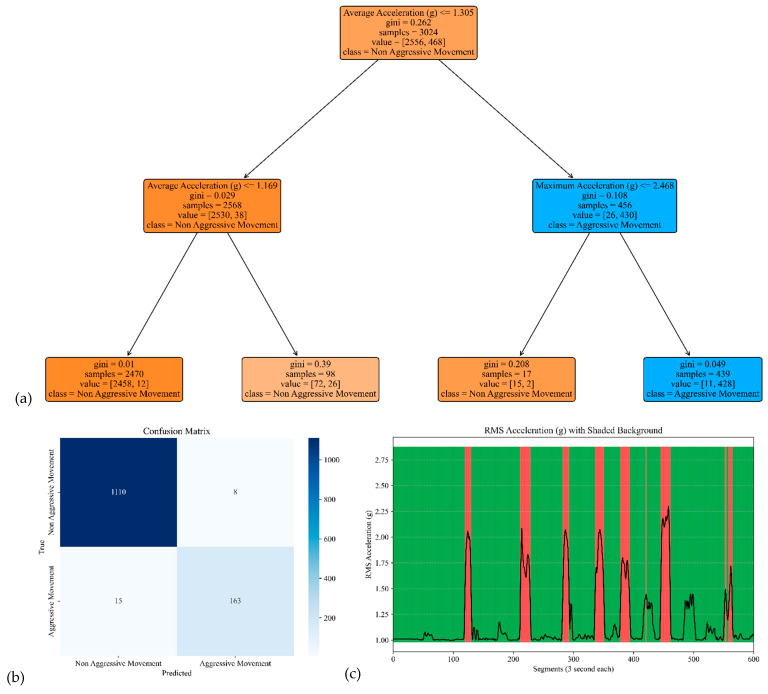
The model performance of the sensor attached to the right leg. (**a**) The analysis identifies pivotal classification features, with the average of the acceleration signal’s norm taking precedence, followed by the maximum of the acceleration’s norm. (**b**) The confusion matrix included True vs. Predicted Negative (Non-Aggressive) and Positive (Aggressive) classes. (**c**) The RMS was estimated for each segment of 3 s of the acceleration’s norm. Red shading represents segments identified as Aggressive Movement, and green shading represents Non-Aggressive Movement.

The model performances for the sensor attached to the left and right legs are shown in Figure 9 and Figure 10. The corresponding model yielded an accuracy, precision, specificity, and sensitivity of 0.98, 0.97, 1.00, and 0.88 for the left leg, respectively, and 0.98, 0.95, 0.99, and 0.92 for the right leg, respectively (see Table 2).

## 4. Discussion

The results of this study demonstrate the feasibility of using IMU sensors and machine learning to detect aggressive shaking movements associated with Shaken Baby Syndrome (SBS). The decision tree model performed highly accurately across all sensor positions, particularly on the head, chest, and limbs, with precision values exceeding 90% and specificity values reaching up to 99%. These results indicate that the model effectively distinguishes between Aggressive and Non-Aggressive Movements, making it a promising tool for real-time SBS detection.

A key advantage of this system is its ability to capture high-resolution motion data, allowing for fine-grained analysis of shaking patterns. Unlike traditional SBS detection methods, such as neuroimaging or video surveillance, IMU-based wearable technology offers a non-invasive, real-time solution that can be deployed in childcare settings or integrated into smart monitoring devices. The ability of the decision tree model to classify movements with minimal computational requirements further supports its feasibility for real-world applications, including deployment on low-power wearable devices.

Another key novelty of this study is the systematic evaluation of six sensor positions—across the head, chest, and limbs—to identify optimal sites for real-time SBS detection from a usability perspective. Head sensors achieved the highest specificity (~0.99) but are less practical for prolonged use due to attachment and comfort challenges. Chest sensors offered comparable accuracy and are more feasible for integration into clothing. Limb sensors, while exhibiting slightly lower sensitivity (~0.85–0.92), perhaps offer the greatest usability. This is supported by Prioreschi et al. (2018), who reported a 94% compliance rate and positive comfort ratings for accelerometer wristbands worn by infants [23]. By comparing multiple body sites within a single experimental framework, this study offers practical guidance for designing SBS detection systems that balance classification performance with real-world applicability.

Despite the strong performance of the model, several limitations warrant further investigation. While the RealCare™ Shaken Baby Doll is widely used in simulation studies, it does not fully replicate the anatomical and physiological characteristics of real infants. In particular, its head-to-body proportions, joint mobility, and soft tissue compliance may differ from those of actual infants, affecting how forces are distributed and absorbed during movement. These biomechanical differences may limit the generalizability of our findings to real-world scenarios. Nonetheless, the doll provides a consistent, ethical platform for developing and validating motion classification models. Future work will focus on enhancing generalizability through studies involving wearable sensors in naturalistic infant care environments.

Furthermore, while this study demonstrated the efficacy of a decision tree model, exploring more complex machine learning architectures, such as deep learning or ensemble methods, may improve classification performance. However, the use of decision tree models in this study was motivated by their low computational complexity, fast inference time, and high interpretability, qualities essential for real-time deployment on wearable devices. Several studies demonstrate that decision trees offer major advantages for on-device deployment on low-power wearables. For example, Sousa et al. achieved 8.3× faster inference on ARM-based MPSoCs with decision trees compared to conventional implementations [24]. In the TinyML domain, lightweight models using decision trees deliver similar accuracy to CNNs or MLPs while using substantially less memory and power [25]. Even hybrid architectures leverage decision trees for efficient filtering, cutting energy costs by nearly 68% compared to running full CNNs [25]. Additionally, the tree structure allows for transparent feature importance assessment and easier debugging, which is advantageous in clinical and regulatory settings. While more complex models may yield marginal performance gains, the balance between accuracy, efficiency, and interpretability makes decision trees a practical choice for the intended application.

Another limitation worth noting is the class imbalance in the dataset, as there were significantly fewer Aggressive Movement segments (749) compared to Non-Aggressive ones (4343). This distribution mirrors real-world conditions, where harmful shaking events are rare relative to routine infant care. To address potential bias toward the majority class, model performance was assessed using multiple metrics, including sensitivity and specificity. As shown in Table 2, the model maintained high sensitivity across sensor positions (ranging from 0.82 to 0.92), demonstrating its ability to detect Aggressive Movements despite the imbalance. Future efforts will focus on incorporating data augmentation techniques or cost-sensitive learning approaches to enhance sensitivity and support more balanced performance in diverse, real-world settings.

In order for this system to transition from a controlled experimental setting to real-world use, the SBS detection algorithm can be integrated into commercially viable infant monitoring platforms. This includes embedding the model within smart wearable devices such as baby socks, wristbands, or onesies that already house sensors for vital sign monitoring. Future clinical validation in home environments and usability testing with caregivers will be essential for regulatory approval, user acceptance, and successful deployment at scale.

Another limitation of this study is the use of eight adult participants in collecting data, chosen to balance practical constraints with the objective of creating a sufficiently large and varied motion dataset. While the number of participants is relatively small, the structured data collection protocol generated over 5000 labeled segments, supporting robust training and validation of the classification model. However, relying on adult volunteers rather than actual caregivers in natural home environments may introduce bias, as simulated shaking and caregiving behaviors might not capture the full range of real-world variability. Future research should aim to include a larger and more diverse cohort, ideally composed of caregivers in naturalistic settings, to better assess generalizability and improve ecological validity.

Overall, this study represents a significant step towards developing an IMU-based, machine-learning-driven system for SBS detection. The results support the feasibility of wearable-based motion analysis as an effective approach for identifying high-risk shaking events. Future work should focus on refining sensor placement strategies, optimizing classification models, and validating performance in real-world caregiving environments. By advancing research in this domain, this technology has the potential to provide an essential tool for preventing SBS-related injuries and improving infant safety.

## Figures and Tables

**Table 1:** Prescribed activities categorized into shaking movements and simulated activities of daily care.

Shaking Movements	
Hold the doll in front with two hands by underarms	Task 1 (no-risk): Gently shake the doll up and down. Task 2 (high-risk): Vigorously shake the doll up and down.
Hold the doll in front with two hands by underarms	Task 3 (no-risk): Lightly shake the doll forward and back. Task 4 (high-risk): Shake the doll forward and back aggressively.
Hold the doll in front with two hands by underarms	Task 5 (no-risk): Slowly shake the doll side to side. Task 6 (high-risk): Quickly shake the doll side to side.
Take the doll from the legs with the head hanging down	Task 7 (high-risk): Aggressively shake the doll
Hold the doll in front by shoulders	Task 8 (high-risk): Aggressively shake the doll by the shoulders.
Hold the doll in front by arms	Task 9 (no-risk): Swing the doll’s arms slowly or playfully. Task 10 (high-risk): Swing the doll’s arms aggressively.
Hold the doll by underarms	Task 11 (no-risk): Throw the doll up in the air and catch playfully.
Hold the doll in front by the underarms	Task 12 (no-risk): Lift and lower the doll above one’s head at a normal speed. Task 13 (high-risk): Lift and lower the doll above one’s head aggressively.
Simulated Activities of Daily Care	
Diaper changing simulation	Task 14 (no-risk): Simulate the diaper-changing process.
Sitting doll on participant’s leg	Task 15 (no-risk): Gently shake the leg while holding the baby by hands. Task 16 (no-risk): Move doll’s arms as if one was gently playing with a baby.
Place the doll against shoulder	Task 17 (no-risk): Perform gentle patting on the doll’s back, mimicking patting to aid with burping.
Two-arm cradling	Task 18 (no-risk): Hold the doll in a cradled position using both arms to capture natural cradling movements.

**Table 2:** Model performance at different sensor positions.

Sensor Positions	Accuracy	Precision	Specificity	Sensitivity
Head	0.97	0.94	0.99	0.86
Chest	0.97	0.96	0.99	0.82
Left Arm	0.97	0.91	0.99	0.85
Right Arm	0.97	0.93	0.99	0.85
Left Leg	0.98	0.97	1.00	0.88
Right Leg	0.98	0.95	0.99	0.92

## Data Availability

The data will be available for non-commercial use upon reasonable request submitted to the corresponding author.
